# Nano-Strategies to Fight Multidrug Resistant Bacteria—“A Battle of the Titans”

**DOI:** 10.3389/fmicb.2018.01441

**Published:** 2018-07-02

**Authors:** Pedro V. Baptista, Matthew P. McCusker, Andreia Carvalho, Daniela A. Ferreira, Niamh M. Mohan, Marta Martins, Alexandra R. Fernandes

**Affiliations:** ^1^UCIBIO, Departamento de Ciências da Vida, Faculdade de Ciências e Tecnologia, Universidade Nova de Lisboa, Caparica, Portugal; ^2^School of Food Science and Environmental Health, College of Sciences and Health, Dublin Institute of Technology, Dublin, Ireland; ^3^Department of Microbiology, Moyne Institute of Preventive Medicine, Schools of Genetics and Microbiology, Trinity College Dublin, University of Dublin, Dublin, Ireland; ^4^Nuritas Limited, Dublin, Ireland

**Keywords:** antimicrobial resistance, multidrug resistance, nanomaterials, nanoparticles, plant-based compounds, novel antimicrobial agents, nanotheranostics

## Abstract

Infectious diseases remain one of the leading causes of morbidity and mortality worldwide. The WHO and CDC have expressed serious concern regarding the continued increase in the development of multidrug resistance among bacteria. Therefore, the antibiotic resistance crisis is one of the most pressing issues in global public health. Associated with the rise in antibiotic resistance is the lack of new antimicrobials. This has triggered initiatives worldwide to develop novel and more effective antimicrobial compounds as well as to develop novel delivery and targeting strategies. Bacteria have developed many ways by which they become resistant to antimicrobials. Among those are enzyme inactivation, decreased cell permeability, target protection, target overproduction, altered target site/enzyme, increased efflux due to over-expression of efflux pumps, among others. Other more complex phenotypes, such as biofilm formation and quorum sensing do not appear as a result of the exposure of bacteria to antibiotics although, it is known that biofilm formation can be induced by antibiotics. These phenotypes are related to tolerance to antibiotics in bacteria. Different strategies, such as the use of nanostructured materials, are being developed to overcome these and other types of resistance. Nanostructured materials can be used to convey antimicrobials, to assist in the delivery of novel drugs or ultimately, possess antimicrobial activity by themselves. Additionally, nanoparticles (e.g., metallic, organic, carbon nanotubes, etc.) may circumvent drug resistance mechanisms in bacteria and, associated with their antimicrobial potential, inhibit biofilm formation or other important processes. Other strategies, including the combined use of plant-based antimicrobials and nanoparticles to overcome toxicity issues, are also being investigated. Coupling nanoparticles and natural-based antimicrobials (or other repurposed compounds) to inhibit the activity of bacterial efflux pumps; formation of biofilms; interference of quorum sensing; and possibly plasmid curing, are just some of the strategies to combat multidrug resistant bacteria. However, the use of nanoparticles still presents a challenge to therapy and much more research is needed in order to overcome this. In this review, we will summarize the current research on nanoparticles and other nanomaterials and how these are or can be applied in the future to fight multidrug resistant bacteria.

## Introduction

Multidrug resistant (MDR) bacteria remain the greatest challenge in public health care. The numbers of infections produced by such resistant strains are increasing globally. This acquired resistance of pathogens presents a key challenge for many antimicrobial drugs. Recent advances in nanotechnology offer new prospects to develop novel formulations based on distinct types of nanoparticles (NPs) with different sizes and shapes and flexible antimicrobial properties.

NPs may offer a promising solution as they can not only combat bacteria themselves but can also act as carriers for antibiotics and natural antimicrobial compounds (Wang et al., 2017a). While various materials have been explored from liposomal to polymer based nano-drug carriers, metallic vectors, such as gold NPs, are attractive as core materials due to their essentially inert and nontoxic nature (Burygin et al., 2009). Arguably the most attractive aspect of NPs drug delivery systems is their ability to introduce a wide range of therapeutics, either bound to their large surface area or contained within the structure, to the site of infection effectively and safely by having a controlled rate of targeted delivery (Pissuwan et al., 2011; Gholipourmalekabadi et al., 2017). By improving the pharmacokinetic profile and therapeutic index of encapsulated drugs compared to free drug equivalents, the dose required to achieve clinical effects can be dramatically decreased (Gao et al., 2018). This in turn, can reduce the toxicity and the adverse side effects associated with high systemic drug concentrations and frequent dosing (Liu et al., 2009).

This review covers the latest approaches in the development of new nanobiotechnology approaches that may challenge the medical practice to fight bacteria and particularly MDR bacteria.

## Nanomaterials against bacteria

Nanomaterials have at least one dimension in the nanometer scale range (1–100 nm) that convey particular physical and chemical properties considerably different from those of bulk materials (Wang et al., 2017a). Among the wide range of nanomaterials, particular interest has been directed toward NPs. NPs have a number of features, which make them favorable as vectors for drugs to combat disease-causing pathogens. These include their enhancement of drug solubility and stability (Huh and Kwon, 2011); their ease of synthesis (Gholipourmalekabadi et al., 2017); their biocompatibility with target agents; and their modulated release, which can be controlled by stimuli, such as light, pH and heat (Wang Z. et al., 2017). Their distinctive functionality in drug delivery is achieved by their ultra-small size and vast surface to volume ratios. This is a key competitive advantage over conventional therapies in the treatment of infections caused by intracellular pathogens and MDR strains. Their functionalization with different (bio)molecules is another important feature. These comprise NPs containing Ag, Au, Al, Cu, Ce, Cd, Mg, Ni, Se, Pd, Ti, Zn, and super-paramagnetic Fe (Hemeg, 2017; Slavin et al., 2017). AgNPs are considered the most effective nanomaterial against bacteria but other metallic NPs, such as CuONPs, TiONPs, AuNPs, and Fe_3_O_2_NPs, have also demonstrated bactericidal effects (Dakal et al., 2016; Hemeg, 2017; Slavin et al., 2017).

While poor membrane transport limits the potency of many antibiotics (Andrade et al., 2013), drug loaded NPs vehicles can enter host cells *via* endocytosis, facilitating their intracellular entry (Wang Z. et al., 2017). Membrane penetration can also be achieved through interactions with surface lipids, for example, using gold NPs in the co-administration of protein-based drugs (Huang et al., 2010). The therapeutic appeal of NPs is enhanced by their ability to confer physical protection against bacterial resistance mechanisms (Huh and Kwon, 2011). Furthermore, the potential to load multiple drug combinations into NPs presents a highly complex antimicrobial mechanism of action, to which, bacteria are unlikely to develop resistance (Huh and Kwon, 2011). Although, this is usually believed to be the case, there are some studies reporting development of bacterial resistance against silver NPs (Panáček et al., 2018). There is also evidence that exposure of bacteria to this type of NPs may increase its antibiotic tolerance (Kaweeteerawat et al., 2017).

**Figure 1:**
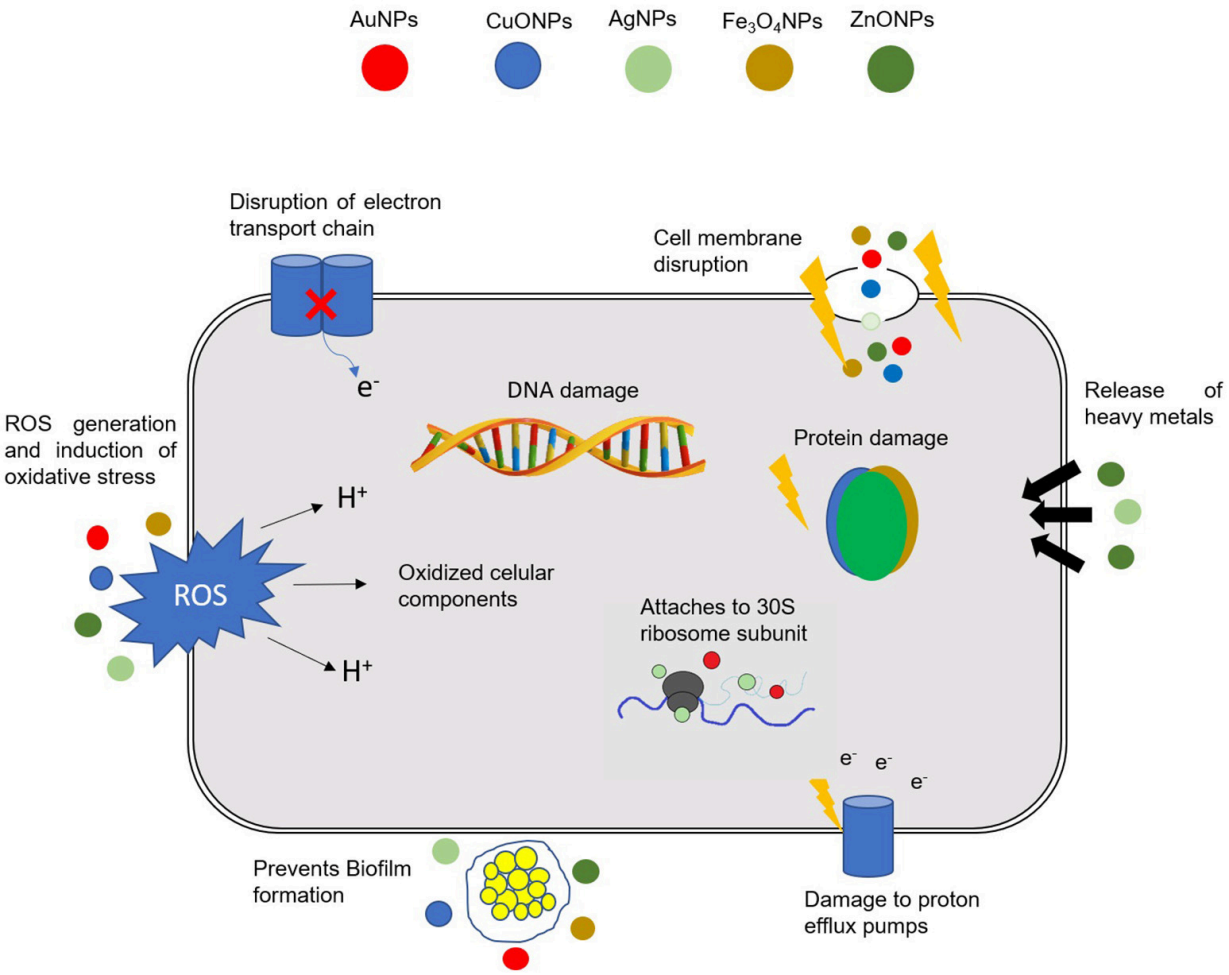
Different mechanisms of action of NPs in bacterial cells. The combination in a single nanomaterial of a multitude of cellular effects may have a tremendous impact in fighting MDR bacteria. DNA, deoxyribonucleic acid; ROS, Reactive oxygen species; AuNPs, gold NPs; CuONPs, Copper oxide NPs; AgNPs, silver NPs; Fe_3_O_4_NPs, iron oxide NPs; ZnONPs, zinc oxide NPs.

NPs can exert their antibacterial activity *via* a multitude of mechanisms, such as: (1) direct interaction with the bacterial cell wall; (2) inhibition of biofilm formation; (3) triggering of innate as well as adaptive host immune responses; (4) generation of reactive oxygen species (ROS); and (5) induction of intracellular effects (*e.g*., interactions with DNA and/or proteins). Because they do not present the same mechanisms of action of standard antibiotics (Figure 1), they can be of extreme use against MDR bacteria (Singh K. et al., 2014; Aderibigbe, 2017; AlMatar et al., 2017; Hemeg, 2017; Natan and Banin, 2017; Rai et al., 2017; Slavin et al., 2017; Zaidi et al., 2017; Bassegoda et al., 2018; Katva et al., 2018; Siddiqi et al., 2018).

Besides the broad-spectrum antibacterial properties that NPs have against Gram-positive and -negative bacteria, NPs have been used as vectors for the delivery of antimicrobial moieties that greatly improve their biocidal properties (Beyth et al., 2015; Rai A. et al., 2016; Singh J. et al., 2016; Esmaeillou et al., 2017; Wang et al., 2017a; Zaidi et al., 2017; Hadiya et al., 2018). Some of the advantages of using NPs as vectors are due to their small and controllable size; their protective action against enzymes that would otherwise destroy antimicrobial compounds; their ability to actively deliver antibiotics; and their capability to combine several therapeutic modalities onto a single nanomaterial (*e.g*., several antibiotics/compounds onto the same NPs for combined action; combining silencing agents and drugs, etc.) (Turos et al., 2007; Huh and Kwon, 2011; Mohammed Fayaz et al., 2011; Liu et al., 2013; Qi et al., 2013; Li et al., 2014; Ranghar et al., 2014; Thomas et al., 2014; Wang et al., 2014; Payne et al., 2016; Rai A. et al., 2016; Singh J. et al., 2016; Yeom et al., 2016; Esmaeillou et al., 2017; Zaidi et al., 2017; Zong et al., 2017; Hadiya et al., 2018).

NPs carriers can tackle bacterial threats “passively,” through prolonged drug retention at the specific infection site, or “actively,” through surface conjugation with active molecules that bind a certain target (Wang Z. et al., 2017). The balance between the surface modification interaction strength, the compound release rate and the stability of the conjugate should be carefully considered for the design of an effective “active” delivery strategy (Burygin et al., 2009; Pissuwan et al., 2011). In an attempt to overcome their therapeutic limitations, various research groups have investigated the conjugation of antibiotics to NPs (Tiwari et al., 2011). For example, Saha et al. describe the direct conjugation of ampicillin, streptomycin and kanamycin to gold NPs (Saha et al., 2007). The resulting complexes were shown to have lower minimum inhibitory concentration (MIC) than the free drug counterparts against both Gram -negative and -positive bacteria. While the detailed mechanism of these effects are not explained by the authors in the above case, Fayaz et al. has attempted to uncover how their vancomycin functionalized gold NPs demonstrated activity against strains which are usually vancomycin resistant based either on mutations (vancomycin resistant *Staphylococcus aureus*), or membrane structure (*Escherichia coli*) (Mohammed Fayaz et al., 2011). They propose that only when the antibiotic was complexed with the NPs could this result in nonspecific, multivalent interactions and anchoring of the carrier to the cell wall synthesis proteins. Based on the presence of pits in the cells, which was observed using transmission electron microscopy, the authors concluded that the consequence of the non-specific binding was compromised membrane integrity, and subsequent cell death (Mohammed Fayaz et al., 2011; Gao et al., 2018).

## Antibacterial mechanism of NPs

The antibacterial activity of NPs against MDR bacteria and biofilms depends on a number of factors, namely, their large surface area in contact with bacteria through electrostatic attraction, van der walls forces or hydrophobic interactions; on the nanoparticle size and stability; together with the drug concentration (Chen et al., 2014; Gao et al., 2014; Li et al., 2015). The interaction of NPs with bacteria generally triggers oxidative stress mechanisms, enzymatic inhibition, protein deactivation and changes in gene expression. Still, the most common antibacterial mechanisms are related to oxidative stress, metal ion release, and non-oxidative mechanisms (Wang et al., 2017a; Zaidi et al., 2017 see Figure 1).

Oxidative stress induced by ROS is one of the most important mechanisms assisting the antibacterial activity of NPs (Dwivedi et al., 2014; Rudramurthy et al., 2016). ROS are natural byproducts of cellular oxidative metabolism and have significant important roles in the modulation of cell survival and death, differentiation and cell signaling. In bacteria, ROS are formed from aerobic respiration, and their production is balanced by the cell antioxidant machinery but upon an additional ROS insult, oxidation of biomolecules, and cell components result in severe cellular damage (Li et al., 2012b). The excessive production of ROS leads to a disturbed redox homeostasis resulting in oxidative stress, affecting membrane lipids and altering the structure of DNA and proteins (Dwivedi et al., 2014). It has been shown that while ${\text{O}}_{2}^{-}$ and H_2_O_2_ can be neutralized by endogenous antioxidants, ·OH and singlet oxygen (^1^[O_2_]) lead to acute microbial death (Zaidi et al., 2017). Different NPs may generate distinctive ROS, such as superoxide (${\text{O}}_{2}^{-}$) or hydroxyl radical (·OH), hydrogen peroxide (H_2_O_2_), and ^1^[O_2_]) (Wang et al., 2017a). In this manner, the level of ROS generated by NPs is dependent on the chemical nature of the NPs themselves. Application of metallic NPs is currently being considered to overcome bacterial infections since they have shown antimicrobial efficacy due to their high surface-to-volume ratio. An increase ratio is usually accompanied by increased production of ROS, including free radicals. Zhang et al. (2013) demonstrated that ROS generation and metal ion release significantly enhanced the antibacterial activity through uncoated AuNPs in aqueous suspension under UV irradiation (365 nm). Umamaheswari (Umamaheswari et al., 2014) demonstrated that the antibacterial activity of AuNPs against *E. coli, Salmonella* Typhi, *Pseudomonas aeruginosa* and *Klebsiella pneumoniae* were due to oxidative stress caused by increased intracellular ROS. A recent study (Zhang et al., 2013) evaluated AuNPs and AuNPs -laser combined therapy against *C. pseudotuberculosis* and suggested that the mechanism of action is related with ROS production, that causes an increase of oxidative stress of microbial cells in the form of vacuole formation as an indication of potent activity. This effect was higher with AuNPs-laser, causing a rapid loss of bacterial cell membrane integrity due to the fact that laser light enhances at least one fold antimicrobial activity of AuNPs. Several other studies have addressed the role of metal NPs to induce MDR bacteria death *via* oxidative stress (Table 1) (Foster et al., 2011; Li et al., 2012b; Rai et al., 2012; Zhang et al., 2013; Reddy L. S. et al., 2014; Singh R. et al., 2014; Pan et al., 2016; Courtney et al., 2017; Ulloa-Ogaz et al., 2017; Zaidi et al., 2017). Indeed, titanium dioxide NPs were shown to adhere to the surface of the bacterial cell and trigger the production of ROS, which in turns lead to damage of the structure of cellular components and consequent cell death (Foster et al., 2011). In another important study using different metal NPs, AgNPs were shown to generate superoxide radicals and hydroxyl radicals, whereas Au, Ni, and Si NPs generated only singlet oxygen, which upon entering the cell produced an antibacterial effect (Zhang et al., 2013). More recently, Reddy and co-workers demonstrated that ZnONPs alone can also act as an effective antibacterial agent *via* the generation of ROS (Reddy L. S. et al., 2014). Exposure to UV irradiation may also potentiate the action of NPs. Li et al. (2012b) reported the augmented antibacterial effects of zinc oxide (ZnO) and titanium oxide (TiO) NPs triggered by UV irradiation as the results of the increased production of superoxide, hydroxyl and singlet oxygen radicals that potentiated bacteria mortality by severe oxidative stress. Graphene oxide–iron oxide NPs have also demonstrated maximum antibacterial activity due to the generation of hydroxyl radicals and diffusion into bacterial cells (Pan et al., 2016). More recently, Ulloa-Ogaz and collaborators demonstrated that copper oxide NPs interact with bacteria, generating an intracellular signaling cascade that trigger oxidative stress and, thus, an antibacterial effect (Ulloa-Ogaz et al., 2017).

**Table 1:** Nanoparticles against MDR pathogens and their mechanisms of action.

**Type of nanoparticles**	**Targeted bacteria**	**Antibiotic resistance type**	**Mechanisms of antibacterial action**	**References**
AgNPs	*Enterococcus faecalis, S. aureus*	Vancomycin-resistant	Combination with vancomycin. Bacterial cell death.	Saeb et al., 2014; Esmaeillou et al., 2017
	*Enterococcus*		On-going investigations.	Percival et al., 2007
	*S. aureus*	Methicillin-resistant	Combination with antibiotics.	Brown et al., 2012; Saeb et al., 2014; Esmaeillou et al., 2017
			Physical adhesion to the bacterial cell.	Su et al., 2011
			On-going investigations.	Percival et al., 2007
	*E. coli, P. aeruginosa*	Ampicillin- resistant	Combination with ampicillin leads to entry into the bacterial cell. Inhibition of cell wall synthesis, protein synthesis and nucleic acid synthesis.	Lara et al., 2010; Brown et al., 2012
	*S. aureus, E. coli, P. aeruginosa, K. pneumoniae, E. faecalis, Salmonella* Typhimurium*, Bacillus cereus*	Erythromycin-resistant	Cell surface damage and loss of the chain integrity.	Otari et al., 2013
	*S. pneumoniae*	Teicoplanin-resistant	ROS generation, cellular uptake of silver ions, cascade of intracellular reaction.	Thapa et al., 2017
	*E. coli, S. aureus*	Ampicillin- resistant		
	*E. coli, S. aureus*	Tetracycline-resistant	Combination with tetracycline.	Djafari et al., 2016
	*P. aeruginosa*	Ofloxacin-resistant	Evade multidrug efflux pumps.	Ding et al., 2018
	*P. aeruginosa, MRSA*, VRE, *Serratia marcescens*	Biofilm formation	Ongoing investigations.	Percival et al., 2007
	*Enterobacter cloacae, S. mutans*		ROS production and membrane disruption.	Kulshrestha et al., 2017
	*S. epidermidis, S. aureus*		Penetration in the bacterial biofilm using an external magnetic field.	Mahmoudi and Serpooshan, 2012
	*E. coli*	MDR	ROS generation.	Zhang et al., 2013; Siddiqi et al., 2018
	*E. coli, P. aeruginosa*			Ramalingam et al., 2016
	*S. aureus, Enterococcus* spp., *P. aeruginosa, A. baumannii, Enterobacteriaceae*		Interaction with components of the cells where chemical and physical properties are modified.	Cavassin et al., 2015
	*E. coli*			Lok et al., 2007
	*S. aureus, E. coli, P. aeruginosa, K. pneumoniae, B. subtilis*		Penetration in the bacterial cell wall.	Acharya et al., 2018
	*P. aeruginosa*		Combined therapy, using blue light.	El Din et al., 2016
	*E. coli, Pseudomonas fluorescens, Pseudomonas putida, P. aeruginosa, B. subtilis, S. aureus*		Disruption of the bacterial cell wall.	Bondarenko et al., 2013
	*A. baumannii*		Attach to the cell wall leading to structural changes in the permeability of the cell membrane.	Chang et al., 2017
	*P. aeruginosa*			Singh K. et al., 2014; Salomoni et al., 2017
	*S. aureus, E. coli*			Jung et al., 2008; Muniyan et al., 2017
	*P. aeruginosa, E. coli*		Combination with antibiotics.	Esmaeillou et al., 2017
	*E. coli*			Karimi et al., 2016
	*E. faecalis*			Katva et al., 2018
	*Salmonella* Typhimurium			McShan et al., 2015
	*Enterobacteriaceae*			Panáček et al., 2016b
	*S. aureus, P. aeruginosa, E. coli*			Panáček et al., 2016a
	*S. aureus, E. coli*		Upregulation of the expression of antioxidant genes and ATP pumps.	Nagy et al., 2011
	*S. epidermidis*	MDR/Biofilm formation	Conjugation with AMP.	Jaiswal et al., 2015
	*Mycobacterium smegmatis*			Mohanty et al., 2013
	*Vibrio fluvialis, P. aeruginosa*			Lambadi et al., 2015
	*B. subtilis, E. coli*			Liu et al., 2013
	*E. coli*			Pal et al., 2016
	*E. coli, Acinetobacter calcoaceticus, Aeromonas bestiarum, B. Subtili, P. fluorescens, Kocuria rhizophila, Micrococcus luteus*			Ruden et al., 2009
AuNPs	*S. aureus*	Vancomycin-resistant	Combination with vancomycin.	Mohammed Fayaz et al., 2011
	*E. faecalis*			Lai et al., 2015
	*S. aureus*	Methicillin-resistant	Photothermal therapy with ROS generation.	Kuo et al., 2009; Millenbaugh et al., 2015; Mocan et al., 2016; Hu et al., 2017; Ocsoy et al., 2017
			Combination with vancomycin.	Lai et al., 2015
	*E. coli, K. pneumoniae*	Cefotaxime-resistant	Disruption of the bacterial cell wall, DNA damage.	Shaikh et al., 2017
	*S. aureus, E. coli, P. aeruginosa, Enterobacter aerogenes*	Ampicillin-resistant	Combination with ampicillin. Lead to entry into the bacterial cell.	Brown et al., 2012
	*Streptococcus bovis, S. epidermidis, E. Aerogenes*	Kanamycin-resistant	Disruption of the bacterial cell wall.	Payne et al., 2016
	*K. 45ellular45, Proteus mirabilis, A. baumannii*	Carbapenems-resistant	Disturb of osmotic balance and disrupt the integrity of cell bacterial cell wall.	Shaker and Shaaban, 2017
	*P. aeruginosa*	Biofilm formation	Interaction with cell surface.	Yu et al., 2016
	*S. aureus*		Laser excitation of the near IR LSPR lead to an efficient photothermal response with efficient killing of bacteria biofilms.	Pallavicini et al., 2014
	*E. coli, P. aeruginosa, S. aureus*		Penetration through biofilm layers and interaction with cellular components.	Ramasamy et al., 2017a,b
	*S. epidermidis, S. haemolyticus*		Combination with antibiotics.	Roshmi et al., 2015
	*Proteus species*		Interaction between proteins and NPs.	Vinoj et al., 2015
	*E. coli, P. aeruginosa, S. aureus, B. Subtilis*		ROS generation.	Wang Z. et al., 2017
	Gram-negative bacteria	MDR	Automated microarray-based system that identifies Gram-negative pathogens from positive blood cultures and resistance mechanism.	Walker et al., 2016
	*S. aureus*		Photoacoustic detection and photothermal therapy	Galanzha et al., 2012
	*E. coli*		ROS generation	Zhang et al., 2013
	*E. coli*		Change of membrane potential and inhibition of ATP synthase; inhibition of the subunit of the ribosome for tRNA binding.	Cui et al., 2012
	*E. coli, K. pneumoniae, S. aureus, B. subtilis*			Shamaila et al., 2016
	*E. coli, K. pneumoniae, E. cloacae*		Photodynamic Therapy/ Photothermal therapy.	Khan et al., 2017
	*S. aureus, E. coli, E. cloacae, P. aeruginosa*			Mocan et al., 2017
	*Salmonella* Typhimurium			Lin and Hamme, 2015
	*S. aureus*			Gil-Tomás et al., 2007
	*E. coli, S. aureus*		Interaction with biomolecules.	Kim D. et al., 2017
	*E. coli, K. pneumoniae*		Not revealed.	Bresee et al., 2014
	*S. aureus, E. coli, P. aeruginosa*		Disruption of bacterial cell wall.	Li et al., 2014; Yang et al., 2017
	*E. coli*		Interaction between lysozyme microbubbles and cell wall.	Mahalingam et al., 2015
	*E. coli, S. aureus, Salmonella* Typhimurium		Depend of co-existing chemicals that were not removed from AuNPs.	Shareena Dasari et al., 2015; Zhang et al., 2015a
	*E. coli, S. aureus, K. pneumoniae*		Combination with antibiotics.	Pradeepa et al., 2016
	*P. aeruginosa*	MDR/Biofilm formation	Conjugation with AMP.	Casciaro et al., 2017
	*Staphylococci, Enterococci* and other bacterial strain			Kuo et al., 2016
	*E. coli, S. aureus, K. pneumoniae, P. aeruginosa*			Rai A. et al., 2016
	*Salmonella* Typhimurium			Yeom et al., 2016
ZnONPs	*K. pneumoniae*	Ampicillin- carbenicillin-resistant	ROS generation and disruption of bacterial cell wall.	Reddy L. S. et al., 2014
	*S. aureus*	Methicillin-resistant	Enzyme inhibition.	Cha et al., 2015
	*E. coli*	*MDR*	ROS generation and disruption of bacterial cell wall.	Li et al., 2012b; Tong et al., 2013; Chakraborti et al., 2014; Gelabert et al., 2016; Nagvenkar et al., 2016
	*B. subtilis*			Hsueh et al., 2015
	*S. aureus*			Lakshmi Prasanna and Vijayaraghavan, 2015; Nagvenkar et al., 2016
	*Vibrio cholerae*			Sarwar et al., 2016
	*S. aureus, E. coli, Proteus, Acinetobacter, P. aeruginosa*		Combination with antibiotics.	Ehsan and Sajjad, 2017
	*S. aureus, E. coli, S. mutants*		Depend on components and structure of the bacteria cell wall.	Yu et al., 2014
	*S. aureus, P. aeruginosa*	Biofilms formation	ROS generation.	Aswathanarayan and Vittal, 2017
	*Streptococcus sobrinus*			Aydin Sevinç and Hanley, 2010
CuONPs	*E. coli, S. aureus*	MDR	ROS generation.	Singh R. et al., 2014; Chakraborty et al., 2015
	*S. aureus, P. aeruginosa*			Ulloa-Ogaz et al., 2017
	*Paracoccus denitrificans*		Modulation of nitrogen metabolism.	Su et al., 2015
	*S. aureus*	Biofilm formation	Ongoing investigations.	Chen et al., 2014
CuNPs	*S. aureus*	Methicillin-resistant	Copper ions release and subsequently bind with DNA leading to disorder of helical structure.	Kruk et al., 2015
	*P. aeruginosa*	Biofilm formation	Penetrate the cell wall and damage the nucleic acid.	LewisOscar et al., 2015
	*P. aeruginosa*	MDR	Generation of Cu hydrosols.	Zhang et al., 2015b
Fe_3_O_4_NPs	*E. coli*	MDR	Radiofrequency (RF) coupled with magnetic core shell nanoparticles lead to RF-mediated physical perturbation of cell membranes and bacterial membrane dysfunction.	Chaurasia et al., 2016
	*S. aureus, P. aeruginosa, E. coli*		Penetrate the membrane and interference in the electron transfer.	El-Zowalaty et al., 2015
	Gram-positive and -negative bacteria		ROS generation.	Behera et al., 2012
	Gram-positive and -negative bacteria		Nanotechnology to capture Gram- positive and -negative bacteria.	Reddy P. M. et al., 2014
	*S. aureus*	Biofilm formation	ROS generation.	Leuba et al., 2013
Al_2_O_3_NPs	*S. aureus*	Methicillin-resistant	Disruption of bacterial cell wall and ROS generation.	Ansari et al., 2013
	*E. coli*	MDR	Penetration and accumulation inside bacterial cell wall.	Ansari et al., 2014
TiO_2_NPs	*S. aureus*	Methicillin-resistant	Release ions and react with thiol group of proteins present on bacteria surface.	Roy et al., 2010
	*E. coli*	MDR	ROS generation and disruption of bacterial cell wall.	Li et al., 2012b
	*E. coli* and Gram-positive bacteria		Photocatalytic disinfection.	Foster et al., 2011
	*E. coli*		Peroxidation and decomposition of membrane fatty acids.	Joost et al., 2015
Cu/Zn bimetal NPs	*S. aureus*	Methicillin-resistant	Membrane disruption, DNA damage, inhibition of protein synthesis.	Ashfaq et al., 2016
Au/Ag bimetallic NPs	*Enterococcus*	Vancomycin-resistant	Theranostic system for SERS and aPDT.	Zhou et al., 2018
	*E. coli, S. aureus, E. faecalis, P. aeruginosa*	Biofilm formation	Disruption of bacterial cell wall and inactivate the proteins and enzymes for ATP production.	Ramasamy et al., 2016
	*B. subtilis E. coli, K. pneumoniae, S. aureus*	MDR	Combination with antibiotics.	Baker et al., 2017
	*P. aeruginosa, E. coli, S. aureus, Micrococcus luteus*			Fakhri et al., 2017
	*E. coli, S. aureus*			dos Santos et al., 2012
Au/Pt bimetallic NPS	*E. coli*	MDR	Damage of the inner membrane, increase intracellular ATP level.	Zhao et al., 2014
Au/ Fe_3_O_4_NPs	*P. aeruginosa*	MDR	Disruption of bacterial cell wall.	Niemirowicz et al., 2014
Cu/Ni bimetallic NPs	*S. aureus, E. coli, S. mutans*	MDR	Adsorption of ions to the bacteria cells.	Argueta-Figueroa et al., 2014
MgF_2_NPs	*S. aureus*	Biofilm formation	Attach and penetrate cell surface leading to disruption in membrane potential, promotes the lipid peroxidation and DNA binding.	Lellouche et al., 2009; Chen et al., 2014
Graphene Oxide NPs	*S. aureus*	Methicillin-resistant	Combine antibiotics with exposure to NIR.	Pan et al., 2016
	*E. coli, E. faecalis*	MDR	UV irradiation lead to generation of ROS.	Govindaraju et al., 2016
	*E. coli, P. aeruginosa, K. pneumoniae, S. aureus*		Multiple toxic mechanisms.	Jankauskaite et al., 2016
	*E. cloacae, S. mutans*	Biofilm formation	ROS generation, release of ions.	Kulshrestha et al., 2017
SeNPs	*S. aureus, E. coli*	MDR	Theranostic nanoplatform for selective imaging and targeted therapy: Disruption of the bacteria cell wall.	Huang et al., 2017
SiNPs	*S. aureus*	Methicillin-resistant	Theranostics nanoprobe for near-infrared fluorescence imaging and photothermal therapy: Disruption of the bacteria cell wall.	Zhao et al., 2017

Metal oxides slowly release metal ions that are up taken by the cell, reaching the intracellular compartment where they can interact with functional groups of proteins and nucleic acids, such as amino (–NH), mercapto (–SH), and carboxyl (–COOH) groups (Wang et al., 2017a). This interaction alters the cell structure, hampers enzymatic activity and interferes with the normal physiological processes in the bacterial cell. It has been shown that copper oxide (CuO) NPs cause a significant alteration of the expression of key proteins and may inhibit denitrification. Proteomic analysis showed that CuONPs cause an alteration of proteins involved in nitrogen metabolism, electron transfer and transport (Su et al., 2015). Also, the interaction of gold–superparamagnetic iron oxide NPs with bacterial proteins *via* disulfide bonds affects the metabolism and redox system of bacterial cells (Niemirowicz et al., 2014). NPs may also enter bacteria through absorption, releasing metal ions to the surrounding medium and/or binding to the negatively charged functional groups of the bacterial cell membrane. For example, silver ions (from silver NPs) are adsorbed on the cell membrane, leading to protein coagulation (Jung et al., 2008). Jankauskaitl and collaborators described the bactericidal effect of graphene oxide/Cu/Ag NPs against *E. coli, P. aeruginosa, K. pneumoniae, S. aureus*, and Methicillin-resistant *S. aureus* (MRSA) through a possible synergy between multiple toxic mechanisms (Jankauskaite et al., 2016).

Non-oxidative mechanisms involve interaction of the NPs with the cell wall. In bacteria, the cell wall and membrane behave as defensive barriers that protect against environmental insults. Cell membrane components provide different adsorption pathways for the NPs (Lesniak et al., 2013). The cell wall of Gram-negative bacteria is composed of lipoproteins, phospholipids and lipid polysaccharides (LPS), which form a barrier only allowing the entry of certain macromolecules (Zaidi et al., 2017). In Gram-positive bacteria, the cell wall is composed of a thin layer of peptidoglycans and abundant pores that allow the penetration of foreign molecules, leading to covalent binding with proteins and cellular components, interrupting the proper functioning of the bacterial cell (Sarwar et al., 2015). In addition, Gram-positive bacteria have a highly negative charge on the surface of the cell wall. For example, LPS provides negatively charged regions on the cell wall of Gram-negative bacteria that attracts NPs; and, since teichoic acid is only expressed in Gram-positive bacteria, the NPs are distributed along the phosphate chain. As such, the antimicrobial effect is more foreshadowed in Gram-positive than -negative bacteria (Wang et al., 2017a). Indeed, Yu and colleagues synthesized a novel hydroxyapatite whisker (HAPw)/zinc oxide (ZnO) NPs and evaluated the antimicrobial effect against *S. aureus, E. coli*, and *Streptococcus mutans*. The authors demonstrate that the antibacterial effect depends on the components and structure of the bacterial cell wall. The antibacterial action of these NPs could be improved for Gram-positive bacteria and certain components could prevent the adhesion of ZnO NPs to the bacterial cell barrier (Yu et al., 2014). Ansari et al. reported that the accumulation on NPs in the bacterial cell wall causes irregularly shaped pit, perforation and disturbs metabolic processes (Ansari et al., 2014). In a study carried out by Joost and co-workers, it was demonstrated that a treatment with TiO_2_ NPs increased the bacterial cell volume, resulting in membrane leakage (Joost et al., 2015).

## Biofilm formation and quorum-sensing

Biofilm formation plays an important role in bacterial resistance protecting bacteria and allowing then to evade the action of antibiotics (Lebeaux et al., 2014; Khameneh et al., 2016). The most active fractions of bacteria are now recognized to occur as biofilms, where cells are adhered to each other on surfaces within a self-produced matrix of extracellular polymeric substance (EPS). EPS provide a barrier allowing to inhibit the penetration of antibiotics and further promote antibiotic resistance leading to a serious health threat worldwide since biofilms are resistant to antibiotics penetration and escape innate immune system by phagocytes (Hall-Stoodley et al., 2004; Bjarnsholt, 2013). Numerous experimental evidence show that NPs are capable of disrupting the bacterial membranes and can hinder biofilm formation thus reducing the survival of the microorganism (Peulen and Wilkinson, 2011; Leuba et al., 2013; Pelgrift and Friedman, 2013; Slomberg et al., 2013; Chen et al., 2014; Miao et al., 2016; Yu et al., 2016; Kulshrestha et al., 2017). This way, NPs provide an alternative strategy to target bacterial biofilms with potential to use both antibiotic-free and antibiotic-coated approaches (Gu et al., 2003; Li et al., 2012a; Sathyanarayanan et al., 2013). Earlier reports demonstrated that NPs are able to interfere with biofilm integrity by interacting with EPS and with the bacterial communication - quorum sensing (QS). The properties of NPs must be designed to be able to inhibit biofilm formation namely through size and surface chemistry. The size of NPs is important to it since they must be able to penetrate the EPS matrix and surface chemistry will command the amount of interactions with the EPS (Lundqvist et al., 2008). The majority of the strategies to achieve inhibition of biofilm formation are to target and interfere with QS molecules (Singh et al., 2017).

QS systems in bacterial populations act as major regulatory mechanisms of pathogenesis, namely in the formation of biofilm structures. These systems help bacteria to “communicate” with each other, through the production and detection of signal molecules (Rutherford and Bassler, 2012; Papenfort and Bassler, 2016). Using this cell-to-cell communication, bacterial populations are able to synchronize the expression of their genes, acquiring competitive advantage to respond to changes in the environment (Rutherford and Bassler, 2012). Therefore, QS systems are known to promote the formation of antibiotic tolerant biofilm communities. It is known that biofilm structures are a recalcitrant mode of bacterial growth that increases bacterial resistance to conventional antibiotics (Reen et al., 2018). This way, bacterial biofilms pose a significant challenge to the efficacy of conventional antibiotics being considered an essential platform for antibiotic resistance (Høiby et al., 2011). Taking this into account, it isn't surprising that the targeting and disruption of QS signaling systems and consequently, of the biofilm production, set the pillar for future next-generation anti-virulence therapies to be developed (LaSarre and Federle, 2013; Venkatesan et al., 2015; Jakobsen et al., 2017).

Surface-functionalized NPs with β-cyclodextrin (β-CD) or N-acylated homoserine lactonase proteins (AiiA) are able to interfere with signaling molecules preventing these molecules from reaching its cognate receptor, therefore inhibiting the signal/receptor interaction. This process will “turn off” QS and obstructing the bacterial communication (Kato et al., 2006; Ortíz-Castro et al., 2008). Several papers reported inhibition of biofilm formation namely by gold NPs (AuNPs). Acyl homoserine lactones (AHL) are signaling molecules with a role in bacterial QS and bind directly to transcription factors to regulate gene expression Recently, Gopalakrishnan and colleges synthesized (Vinoj et al., 2015) AuNPs coated AiiA purified from *Bacillus licheniformis*. These AiiA AuNPs inhibited EPs production and demonstrated potent antibiofilm activity against Proteus species at 2–8 μM concentrations without being harmful for the host cells at the 2μM concentration. Sathyanarayanan et al. (2013) demonstrated that using AuNPs there is a significant reduction of *S. aureus* and *P. aeruginosa* biofilms applied in high concentration (exceeding 50 mg/L). A recent study by Yu et al. (2016) demonstrated that AuNPs were able to strongly attenuate biofilm formation of *P. aeruginosa*. The inhibition observed in this study was related with interruption of adhesin- mediated interaction between the bacteria and the substrate surface due to electrostatic attractions between the AuNPs and cell wall surface of *P. aeruginosa*, instead of QS-related molecules. Positive charge AuNPs inhibited significantly *S. aureus* and *P. aeruginosa* biofilm formation (while minimizing mammalian cytotoxicity) (Ramasamy et al., 2016). The use of NPs demonstrates an exclusive approach to penetrate infectious biofilms and target bacterial communication, overcoming this major health issue related with biofilm infections.

Because most of these NPs-based platforms exert their action *via* distinct mechanisms/structures/pathways of those used by traditional antibiotics, combined therapeutic regimens are promising strategies to tackle the surge of multidrug resistant (MDR) bacteria bypassing their defense mechanisms (Pelgrift and Friedman, 2013; Singh K. et al., 2014; Hemeg, 2017; Zaidi et al., 2017). Additionally, NPs have been shown to activate macrophages in a dose dependent manner (Patel and Janjic, 2015) which promotes the host defenses (Hemeg, 2017; Jagtap et al., 2017).

This multi-target action of NPs may overcome multidrug resistance by circumventing several obstacles encountered by traditional antibiotics (Pelgrift and Friedman, 2013; Chen et al., 2014; Singh K. et al., 2014; Hemeg, 2017; Jagtap et al., 2017; Rai et al., 2017; Zaidi et al., 2017). Table 1 highlights several types of NPs that have shown effective bactericidal activity when administered isolated; combined with standard antibiotics; and/or radiation or as vectors for biocidal delivery allowing killing of MDR bacteria, and in some cases also inhibiting biofilm production.

We will now focus on the different types of metal NPs highlighting their most relevant mechanism/effects against MDR bacteria and/or biofilms structures.

## Silver nanoparticles (AgNPs)

Since the ancient times, silver has been recognized as having antimicrobial effects (Rai et al., 2009; Reidy et al., 2013). Based on all the evidence to date, AgNPs are probably one of the most promising inorganic NPs that can be used for the treatment of bacterial infections (Natan and Banin, 2017). These NPs may be synthesized by traditional chemical reduction or *via* “green” chemistry approaches using plant and/or microbial extracts (Iravani et al., 2014; Ribeiro et al., 2018).

Several mechanisms have been proposed to understand how AgNPs mediate cell death, including cell wall disruption (Lok et al., 2007; Bondarenko et al., 2013), oxidation of cellular components, inactivation of the respiratory chain enzymes, production of ROS, and decomposition of the cellular components (Chen et al., 2014; Rizzello and Pompa, 2014; Dakal et al., 2016). The permeability of the membrane increases after incorporation of AgNPs into the cell membrane. The adsorption of the NPs leads to the depolarization of the cell wall, altering the negative charge of the cell wall to become more permeable. It was demonstrated disruption of the cell wall with subsequent penetration of the NPs. The entry of AgNPs induces ROS that will inhibit ATP production and DNA replication (Zhang et al., 2013; Dakal et al., 2016; Durán et al., 2016; Ramalingam et al., 2016). However, there is evidence that AgNPs can release Ag^+^, known to exhibit antimicrobial activity, when interacting with thiol-containing proteins, which weaken their functions (Durán et al., 2010). The precise method of the antibacterial mechanism of AgNPs is still not completely understood (Franci et al., 2015; Durán et al., 2016). All the existing data indicates that AgNPs exert several bactericidal mechanisms in parallel, which may explain why bacterial resistance to silver is rare (Karimi et al., 2016). Concerns regarding the cytotoxicity and genotoxicity of AgNPs have been raised (Chopra, 2007) but various authors have conducted clinical trials based on AgNPs and no important clinical alterations have been detected (Munger et al., 2014a,b; Smock et al., 2014). Interestingly, AgNPs have been found to exhibit higher antimicrobial activity than antibiotics like gentamicin or vancomycin against *P. aeruginosa* and MRSA (Saeb et al., 2014). Lara *et al*. showed the potential bactericidal effect of AgNPs against MDR *P. aeruginosa*, ampicillin-resistant *E. coli* O157:H7 and erythromycin-resistant *Streptococcus pyogenes* (Lara et al., 2010). Nagy *et al.*, reported that AgNPs were capable of inhibiting the growth of *S. aureus* and *E. coli via* the up-regulation of the expression of several antioxidant genes and ATPase pumps (Nagy et al., 2011). Dolman *et al*. also showed that the Ag-containing Hydrofiber® dressing and nanocrystalline Ag-containing dressing are effective agents against antibiotic sensitive Gram-negative and -positive bacteria as well as antibiotic resistant bacteria, such as MRSA, Vancomycin-resistant *Enterococci* (VRE) and *Serratia marcescens*, avoiding the formation of biofilms on biomaterials (Percival et al., 2007). Su and collaborators showed that AgNPs immobilized on the surface of nanoscale silicate platelets (AgNP/NSPs) have strong antibacterial activity against MRSA and silver-resistant *E. coli via* generation of ROS (Su et al., 2011). Singh and collaborators showed that AgNPs from *P. amarus* extract exhibited excellent antibacterial potential against MDR strains of *P. aeruginosa* (Singh K. et al., 2014). Recently, two different shaped AgNPs (spheres and rods) were used against Gram-positive and -negative bacteria, both showing promising antibacterial activity against different strains (Acharya et al., 2018).

An emerging practice is to combine AgNPs with antibiotics to enhance antimicrobial potency. Recently, Katya and collaborators showed that the combination of gentamicin and chloramphenicol with AgNPs has a better antibacterial effect in MDR *E. faecalis* than both antibiotics alone (Katva et al., 2018). McShan *et al*. described that AgNPs combined with either one of two-different class of antibiotics (tetracycline and neomycin) can exhibit a synergistic effect, showing an enhanced antibacterial activity at concentrations below the MIC of either the NPs or the antibiotic (McShan et al., 2015). Other authors also reported similar results (Thomas et al., 2014; Panáček et al., 2016a,b; Salomoni et al., 2017). Djafari and collaborators described the synthesis of water-soluble AgNPs using the antibiotic tetracycline as co-reducing and stabilizing agent (AgNPs@TC) and demonstrated their effectiveness against tetracycline-resistant bacteria (Djafari et al., 2016).

Antimicrobial peptides (AMPs) represent one of the forms of defense strategy against infections in living organisms and are emerging as essential tools to kill pathogenic bacteria, since they exhibit broad-spectrum activity and low resistance development (Yeaman, 2003). Lytic peptides are AMPs produced by all organisms. In mammals, they are an innate host defense mechanism against pathogens (Bahar and Ren, 2013). The mechanism of action of AMPs relies on the ability to interact with bacterial membranes or the cell wall, thus inhibiting cellular biochemical pathways and ultimately killing the bacteria (Zhang and Gallo, 2016). Defensins and cathelicidin are two of the larger families of lytic peptides that kill bacteria by disrupting the membrane. Unfortunately, AMPs have poor enzymatic stability, low permeability across biological barriers and may be rapidly degraded in the human body by proteases, which greatly limits their application (Wang, 2014). Immobilization of the peptides onto NPs can increase their stability, enhancing the antimicrobial properties of the NPs and therefore, has the potential to be used as a new tool to tackle antibiotic resistant bacteria (Brandelli, 2012; Rai A. et al., 2016). Indeed, the first author to demonstrate that functionalized AgNPs with peptides increased their antibacterial activity was Ruden and co-workers (Ruden et al., 2009). Based on this strategy several researchers functionalized AgNPs with AMPs (AgNP@AMP) with increases in the antimicrobial activity compared with free AMPs (Ruden et al., 2009; Liu et al., 2013; Mohanty et al., 2013). Polymyxin B is the most used AMP and exhibits antibacterial activity *via* interaction with the endotoxin LPS in the outer membrane of Gram-negative bacteria (Morrison and Jacobs, 1976; Lambadi et al., 2015). It was proved that AgNPs functionalized with polymyxin-B removed almost completely endotoxins from solutions and hindered the formation of biofilm onto surgical blades (Jaiswal et al., 2015; Lambadi et al., 2015). Liu *et al.*, demonstrated that the immobilization of peptides with AgNPs enhanced their antimicrobial activity compared to an unbound peptide and also minimized toxicity of AgNPs compared to using the AgNPs alone (Liu et al., 2013). A recent study by Pal *et al*. describes a system consisting of a cysteine containing AMP conjugated with AgNPs, which demonstrated that the Ag-S bonds increased stability and enhanced antimicrobial activity than conjugation using electrostatic interactions (Pal et al., 2016).

Other methods have been used to improve the antibacterial activity of AgNPs. One of these methods relies on the use of visible blue light, which was previously shown to exhibit strong antibacterial activity (Dai T. et al., 2013; Maclean et al., 2014). El Din and collaborators demonstrated that blue light combined with AgNPs exhibits therapeutic potential to treat MDR infections and can represent an alternative to conventional antibiotic therapy, since the antimicrobial activity of the combination was greater than the components alone. Moreover, this approach proved to be synergistic in the treatment of an unresponsive antibiotic-resistant bacteria responsible for a wound in a horse (El Din et al., 2016). Spherical shaped thioglycolic acid-stabilized AgNPs (TGA-AgNPs) conjugated with vancomycin were used as drug delivery systems and demonstrated to possess increased antimicrobial activity against MDR bacteria such as MRSA and VRE (Esmaeillou et al., 2017).

## Gold nanoparticles (AuNPs)

Metallic gold is considered inert and non-toxic, which may vary when it shifts form metallic bulk to oxidation states (I and II) (Merchant, 1998). Gold NPs (AuNPs) may be synthesized by traditional chemical reduction of a gold salt or *via* “green” chemistry approaches using plant and/or microbial extracts (Shah et al., 2014). The most used and described method is the chemical synthesis based on the reduction of chloroauric acid by citrate (Lee and Meisel, 1982; Fernandes and Baptista, 2017). Some studies have addressed the potential of using AuNPs as antibacterial agents, but some controversy still exists (Cui et al., 2012; Bresee et al., 2014; Shah et al., 2014; Shareena Dasari et al., 2015; Zhang et al., 2015a; Shamaila et al., 2016).

According to Yu H and collaborators, AuNPs are usually not bactericidal at low concentrations and weakly bactericidal at high concentrations (Shareena Dasari et al., 2015; Zhang et al., 2015a). This is possibly due to the effect of co-existing chemicals, such as gold ions, surface coating agents, and chemicals involved in the synthesis that were not completely removed (Shareena Dasari et al., 2015; Zhang et al., 2015a). However, other authors describe that the antibacterial mechanism of AuNPs is associated to (i) the collapse in the membrane potential, hindering ATPase activity causing a deterioration of the cell metabolism; (ii) hindering of the binding subunit of the ribosome to tRNA (Cui et al., 2012); and (iii) Shamaila and co-workers showed that AuNPs may affect the bacterial respiratory chain by attacking nicotinamide (Shamaila et al., 2016). Since AuNPs are non-toxic to the host (Conde et al., 2014; Li et al., 2014; Rajchakit and Sarojini, 2017), the possibility of fine tuning their conjugation chemistry to act as carriers or delivery vehicles of antibiotics or other antibacterial moieties may enhance their bactericidal effect and potentiate the effect of antibiotics (Zhao and Jiang, 2013; Conde et al., 2014; Li et al., 2014; Uma Suganya et al., 2015; Zhang et al., 2015a; Fernandes et al., 2017).

Cationic and hydrophobic functionalized AuNPs were shown to be effective against both Gram-negative and -positive uropathogens, including MRSA. These AuNPs exhibited low toxicity to mammalian cells (biocompatibility) and the development of resistance to these NPs was very low (Li et al., 2014). Vinoj *et al*. demonstrated that coating AuNPs with N-acylated homoserine lactonase proteins (AiiA AuNPs) resulted in a nanocomposite with activity against MDR species compared with AiiA proteins alone (Vinoj et al., 2015). Other approaches were also studied, as adsorbing AuNPs to PVA-lysozyme micro bubbles potentiate the antibacterial activity due to the interaction of AuNPs with cells membranes causing bacterial lysis (Mahalingam et al., 2015). Galic acid capped AuNPs have also been found to be active against Gram-negative and -positive bacteria (Kim D. et al., 2017). Recently, Ramasamy and collaborators described the direct one-pot synthesis of cinnamaldehyde immobilized on gold nanoparticles (CGNPs) with effective biofilm inhibition of more than 80% against Gram-positive bacteria (methicillin-sensitive and -resistant strains of *S. aureus*, MSSA and MRSA, respectively) and Gram-negative (*E. coli* and *P. aeruginosa*) *in vitro* and *in vivo* (Ramasamy et al., 2017a,b). Also, the integration of AuNPs with ultrathin graphitic carbon nitride was described as having high bactericidal performance against both MDR Gram-negative and -positive bacteria, and a high effectiveness in eliminating existing MDR-biofilms and preventing the formation of new biofilms *in vitro* (Wang Z. et al., 2017). Also, conjugation of antibiotics to AuNPs, such as vancomycin, methicillin, etc., increases their intrinsic activity against MDR strains (Mohammed Fayaz et al., 2011; Lai et al., 2015; Roshmi et al., 2015; Payne et al., 2016). Recently Payne and collaborators develop a single-step synthesis of kanamycin-capped AuNPs (Kan-AuNPs) with high antibacterial activity against both Gram-positive and -negative bacteria, including kanamycin resistant bacteria. The authors observed a significant reduction in the MIC against all the bacterial strains tested for Kan-AuNPs when compared to the free drug. This higher efficacy was due to the disruption of the bacterial envelope, resulting in leakage of the cytoplasmic content and consequent cell death (Payne et al., 2016). Pradeepa and collaborators synthesized AuNPs with bacterial exopolysaccharide (EPS) and functionalized them with antibiotics (levofloxacin, cefotaxime, ceftriaxone and ciprofloxacin). They observed that these AuNPs exhibited excellent bactericidal activity against MDR Gram-positive and -negative bacteria compared to free drugs. *E. coli* was the most susceptible MDR bacteria followed by *K. pneumoniae* and *S. aureus* (Pradeepa et al., 2016). Recently, Yang and collaborators described the effect of small molecule (6-aminopenicillanic acid, APA)-coated AuNPs to inhibit MDR bacteria (Yang et al., 2017). They doped AuNPs into electrospun fibers of poly(ε-caprolactone) (PCL)/gelatin to produce materials that avoid wound infection by MDR bacteria and demonstrated *in vitro* and *in vivo* that APA-AuNPs reduced MDR bacterial infections (Yang et al., 2017). Shaker and Shaaban evaluated the surface functionalization of AuNPs with carbapenems [imipenem (Ipm) and meropenem (Mem)] as a delivering strategy against carbapenem resistant Gram-negative bacteria isolated from an infected human. Both Ipm-AuNPs and Mem-AuNPs, with 35 nm diameter showed a significant increase in antibacterial activity against all the tested isolates (Shaker and Shaaban, 2017). Also, Shaikh and collaborators described recently the synthesis and characterization of cefotaxime conjugated AuNPs to target drug-resistant CTX-M-producing bacteria. The authors could invert resistance in cefotaxime resistant bacterial strains (i.e., *E. coli* and *K. pneumoniae*) by using cefotaxime-AuNPs. Their results reinforce the efficacy of conjugating an unresponsive antibiotic with AuNPs to restore its efficacy against otherwise resistant bacterial pathogens (Shaikh et al., 2017).

Combination of AuNPs with other approaches has also been demonstrated. Indeed, one of the most extraordinary properties of AuNPs is the capability to transform light into heat under laser irradiation (Mendes et al., 2017; Mocan et al., 2017). This property is extremely important because it can be exploited to develop photothermal nanovectors to destroy MDR bacteria at a molecular level (for a complete review see Mocan et al., 2017). For example, Khan and collaborators showed that the combination of Concanavalin-A (ConA) directed dextran capped AuNPs (GNPDEX-ConA) conjugated with methylene blue (MB) (MB@GNPDEX-ConA) and photodynamic therapy (PDT) enhanced the efficacy and selectivity of MB induced killing of MDR clinical isolates, including *E. coli, K. pneumoniae*, and *E. cloacae* (Khan et al., 2017). Gil-Tomas and collaborators described that the functionalization of AuNPs covalently with toluidine blue O–tiopronin forms an enhanced, exceptionally potent antimicrobial agent when activated by white light or 632 nm laser light (Gil-Tomás et al., 2007). Hu and collaborators prepared a mixed charged zwitterion-modified AuNPs consisting of a weak electrolytic 11-mercaptoundecanoic acid (HS-C10-COOH) and a strong electrolytic (10-mercaptodecyl)trimethylammonium bromide (HS-C10-N4) that exhibited *in vivo* and under near-infrared (NIR) light irradiation an enhanced photothermal ablation of MRSA biofilm with no damage to the healthy tissues around the biofilm (Hu et al., 2017). Also, the antibacterial activity of glucosamine-gold nanoparticle-graphene oxide (GlcN-AuNP-GO) and UV-irradiated GlcN-AuNP-GO was evaluated against *E. coli* and *E. faecalis*. Results show that UV irradiation of GlcN-AuNP-GO results in higher antibacterial activity than the standard drug kanamycin (Govindaraju et al., 2016). Ocsoy *et al*. reported the development of DNA aptamer-functionalized AuNPs (Apt@AuNPs) and gold nanorods (Apt@AuNRs) for inactivation of MRSA with targeted PTT (Ocsoy et al., 2017). The authors showed that although both NPs could specifically bind to MRSA cells, Apt@AuNPs and Apt@AuNRs increased resistant cell death for 5% and for more than 95%, respectively through PTT. This difference in induction of cell death was based on the relatively high longitudinal absorption of NIR radiation and strong photothermal conversion capability for the Apt@AuNRs compared to the Apt@AuNPs. However, with the new developments of using AuNPs for hyperthermia in the visible light (Mendes et al., 2017) might additionally potentiate the Apt@AuNPs results observed for these authors (Ocsoy et al., 2017). Recently, Mocan *et al*. also described the synthesis of AuNPs by wet chemistry, their functionalization with IgG molecules following laser irradiation. Their results indicate that administration of IgG-AuNPs following laser irradiation provided an extended and selective bacterial death in a dose dependent manner (Mocan et al., 2016).

In recent years, a new approach relying on the conjugation of AuNPs with AMPs has shown interesting results (Rajchakit and Sarojini, 2017). Indeed, Kuo and collaborators mixed synthetic-peptides containing arginine, tryptophan and cysteine termini [(DVFLG)2REEW4C and (DVFLG)2REEW2C], with aqueous tetrachloroauric acid to generate peptide-immobilized AuNPs [i.e., (DVFLG)2REEW4C-AuNPs and (DVFLG)2REEW2C-AuNPs] that were effective antibacterial agents against *Staphylococci, Enterococci*, and antibiotic-resistant bacterial strains (Kuo et al., 2016). Rai and co-workers demonstrated that the use of cecropin-melittin (CM-SH) a known peptide with antibacterial properties (Boman et al., 1989), functionalized in the surface of AuNPs through Au-S bond, showed higher antimicrobial activity and higher stability in media compared with an *in vitro* and *in vivo* infection animal model with the MIC of CM-SH AuNPs four times lower than free CM-SH (Rai A. et al., 2016). Conjugation of AMP with AuNPs usually involves the formation of the Au-S coordinate covalent bond, relying on the amine or thiol groups in peptides or conjugating specific linkers to AMPs with a terminal (N- or C-terminal) cysteine which helps conjugation with gold (Tielens and Santos, 2010; Xue et al., 2014). However, there is one example where covalent conjugation of an AMP to AuNPs has been achieved *via* Au-O bond (Lai et al., 2015). Other approaches use a linker for the conjugation to AuNPs, Poly(ethylene glycol) carboxylic acid (PEGCOOH) covalently bound to AMP showed a significantly increase of the antibacterial and anti-biofilm activity for resistant Gram-negative bacteria (Casciaro et al., 2017). Yeom and co-workers demonstrated the most advanced *in vivo* clinical application for AuNPs@AMP using infected mice and resulting in the inhibition of *Salmonella* Typhimurium colonization in the organs of the animals (Yeom et al., 2016). The reason behind the increased antimicrobial activity of AuNPs@AMP over the free components is that AuNPs can get a higher concentration of the antibiotic at the site of action. These NPs can interact with LPS, proteins in the membrane of the bacteria and in some cases, penetrate the bacterial membrane through the porin channel. This way they can interact with the inner membrane making the AuNPs@AMP conjugate more efficient than the non-conjugated form (Katz and Willner, 2004; Wangoo et al., 2008; Chen J. et al., 2009).

## Bimetallic NPs

Ag and Au may be used in a single NP to enhance the effects of a drug and reduce the required dose. Alternatively, they can be used alone since they possess antimicrobial properties that are enhanced when combined in the form of bimetallic NPs (Arvizo et al., 2010; Singh R. et al., 2016). The role of Ag against MDR pathogens has been previously described. However, AgNPs are difficult to functionalize with biomolecules and drugs. Such limitation may be circumvented by means of alloy/bimetallic NPs that excel their monometallic counterparts providing improved electronic, optical and catalytic properties (Cho et al., 2005; Shah et al., 2012). As reported above, AuNPs constitute good vectors to the delivery of pharmacologic compounds. Gold(Au)-silver(Ag) alloys are an optimal solution since they combine the antimicrobial effect of silver with the ease of functionalization and improved stability in complex biological media provided by gold (Doria et al., 2010; dos Santos et al., 2012). Fakhri and co-workers synthetized and functionalized AgAuNPs with a tetracycline and concluded that there exists a synergetic effect of the antibiotic with the bimetallic nanoparticle, with greater bactericidal activity of this form in detriment of its free forms. The mechanism of action was established as being the generation of ROS (Fakhri et al., 2017). Also recently, Baker and collaborators described the synthesis and antimicrobial activity of bimetallic AgAuNPs from the cell free supernatant of *Pseudomonas veronii* strain AS41G inhabiting *Annona squamosa L*. The authors showed their synergistic effect with standard antibiotics with 87.5, 18.5, 11.15, 10, 9.7, and 9.4% fold increased activity with bacitracin, kanamycin, gentamicin, streptomycin, erythromycin and chloramphenicol, respectively, against bacitracin resistant strains of *Bacillus subtilis, E. coli*, and *K. pneumoniae* (Baker et al., 2017). Zhao and collaborators have demonstrated the antibacterial activity of AuPtNPs bimetallic NPs against sensitive and drug-resistant bacteria *via* the dissipation of the bacterial membrane potential and the elevation of adenosine triphosphate (ATP) levels (Zhao et al., 2014).

Other types of bimetallic NPs have been studied and their antibacterial activity explored, but in most cases as coating agents and not as a delivery approach and antibacterial activity (Argueta-Figueroa et al., 2014).

## Metal oxides

Metal oxides NPs are among one of the most explored and studied family of NPs and are known to effectively inhibit the growth of a wide range of sensitive and resistant Gram-positive and -negative bacteria, emerging as hopeful candidates to challenge antimicrobial resistance (Raghunath and Perumal, 2017; Reshma et al., 2017; Kadiyala et al., 2018). Iron oxide (Fe_3_O_4_), Zinc oxide (ZnO), and Copper oxide (CuO) possess antimicrobial properties and can be applied in clinical care (Sinha et al., 2011). Due to the intrinsic photocatalytic activity of the metal oxides they generate ROS and become powerful agents against bacteria (Tong et al., 2013; Singh R. et al., 2014). These will be described in more detail on the following sections.

## Iron oxide (Fe_3_O_4_)

The synthesis of iron oxide NPs may be achieved *via* different routes (Babes et al., 1999; Berry and Curtis, 2003). The antibacterial mechanism of these NPs is mainly attributed to dissolved metal ions and the generation of ROS (Wang et al., 2017a). It was shown that superparamagnetic iron oxide NPs interact with microbial cells by penetrating the membrane and interfering with the electron transfer (Behera et al., 2012; El-Zowalaty et al., 2015). Additionally, it has been described that iron oxide NPs can damage macromolecules, including DNA and proteins, through the formation of ROS (Leuba et al., 2013). Pan *et al*. developed a system of reduced graphene oxide (rGO)-iron oxide nanoparticles (rGO-IONP) by the chemical deposition of Fe^2+^/Fe^3+^ ions on nanosheets of rGO in aqueous ammonia. The *in vivo* results showed maximum antibacterial activity due to the generation of hydroxyl radicals that can cause physical and chemical damage, which inactivated MRSA (Pan et al., 2016).

## Zinc oxide (ZnO)

ZnO NPs are often used to restrict microorganism growth, being effective against planktonic bacteria, and also inhibiting the formation of biofilms (Hsueh et al., 2015; Sarwar et al., 2016) (Espitia et al., 2012). These NPs can be synthesized by various methods, from green chemistry to sonochemistry (Salem et al., 2015; Ali et al., 2016; Nagvenkar et al., 2016). The antibacterial mechanism of the NPs is partially attributed to two principal factors, the dissolution of metal ion and the generation of ROS (Gelabert et al., 2016; Nagvenkar et al., 2016; Sarwar et al., 2016). ZnO releases Zn^2+^ in liquid medium and is adsorbed on the surface of bacteria or may entry the cell, where it interacts with functional groups in proteins and nucleic acids, hindering enzyme activity and the normal physiological processes (Yu et al., 2014). However, some authors demonstrated that Zn ions have little antimicrobial activity, implying that dissolution of Zn^2+^ might not be the main mechanism of action (Aydin Sevinç and Hanley, 2010). Sarwar and co-workers demonstrated that nanosized ZnO caused significant oxidative stress to *Vibrio cholera*, the damage inflicted was DNA degradation, protein leakage, membrane depolarization and fluidity (Sarwar et al., 2016). Ehsan and Sajjad, described that ZnO NPs impregnated with antibiotics showed good antibacterial activities against *S. aureus, Proteus, Acinetobacter, P. aeruginosa*, and *E. coli*, being that these were resistant to antibiotics but became sensitive in the presence of these NPs with antibiotics (Ehsan and Sajjad, 2017). It was also discovered that these NPs induce the production of ROS even in the dark, and this happens due to the surface defects on the NPs. The different shapes function as enzyme inhibitors, where nanopyramids are the most effective (Cha et al., 2015; Lakshmi Prasanna and Vijayaraghavan, 2015). Recently, Aswathanarayan and Vittal described the antimicrobial effect of ZnO NPs against MDR Gram-positive and -negative pathogens in comparison to gold and iron NPs and these could be used at concentrations less toxic to mammalian cells (Aswathanarayan and Vittal, 2017). ZnO NPs are also known for inhibiting biofilm formation and production of quorum-sensing-dependent virulence factors in *P. aeruginosa* (Lee et al., 2014; García-Lara et al., 2015).

## Copper oxide (CuO)

Copper containing NPs have been shown effective against animal and plant pathogens (LewisOscar et al., 2015), impeding formation of MDR biofilms, and showing the potential to serve as antimicrobial coating agents (LewisOscar et al., 2015). Kruk *et al*. and Zhang *et al*. showed that copper NPs are capable of inhibiting the growth of MDR bacteria, namely, *P. aeruginosa* and MRSA (Zhang et al., 2014, 2015b; Kruk et al., 2015). The antimicrobial activity of these NPs is comparable to that of AgNPs but at a lower cost (Kruk et al., 2015). Copper oxide NPs generate ROS that often leads to chromosomal DNA degradation, which seems to highlight a “particle-specific” action rather than resulting from the release of metallic ions (Chakraborty et al., 2015). Su and collaborators investigated the effects of CuONPs on bacterial denitrification and explored the effect on the expression of intracellular proteins. When CuONPs entry into bacteria metabolic functions are affected, such as active transport, electron transfer, and nitrogen metabolism (Su et al., 2015).

NPs can also be complexed with other metals, like gallium. Gallium NPs have been described to facilitate phagosome maturation of macrophages infected with virulent *M. tuberculosis* and therefore being able to inhibit growth of this pathogen (Choi et al., 2017).

## The potential for nanotheranostics

NPs applications in biodetection is huge and more insights on pathogen detection using NPs platforms can be seen in Veigas et al. (2013, 2014, 2015); Costa et al. (2014); Weng et al. (2015); Kim J. et al. (2017); Wang et al. (2017b); Galvan and Yu (2018), and Yang et al. (2018).

Theranostics is a combination of diagnosis and therapy onto a single platform, which allow for timely biodetection and/or real-time monitoring of therapy. By using NPs, this can be translated to the nanoscale—Nanotheranostics. NPs have been applied for multiplex high-throughput diagnostics to assist precision therapy. For example, Verigene® is an AuNPs test commercialized for diagnosis. It is an automated microarray-based system that identifies Gram-negative pathogens from positive blood cultures. Verigene® BC-GN also detects key resistance mechanisms (Walker et al., 2016; Claeys et al., 2018). Others have used, magnetic and functionalized magnetic iron oxide NPs as affinity probes to capture Gram-positive and -negative bacteria. The analyses of captured bacteria using matrix-assisted laser desorption/ionization mass spectrometry was <1 h (Reddy P. M. et al., 2014). One pioneer work on nanotheranostics against bacterial infection was the development of a method for *in vivo* photoacoustic detection and photothermal eradication of *S. aureus*. Two-color gold and multilayer magnetic nanoparticles were functionalized with an antibody cocktail for the targeting *of S. aureus*. These platform demonstrated ultrasensitive detections for circulating bacterial cells (CBCs), *in vivo* magnetic enrichment and PT eradication of CBCs (Galanzha et al., 2012). Recently, Zhou and collaborators developed a silicon 2,3-naphthalocyanine dihydroxide (Nc) and Vancomycin functionalized silica-encapsulated, silver-coated gold NPs (Au@AgNP@SiO2@Nc-Van) as a novel theranostic system for surface-enhanced Raman scattering (SERS) detection and antimicrobial photodynamic therapy (aPDT) of vancomycin (Van)-resistant enterococci (VRE) strains (Zhou et al., 2018). These authors observed a 4–5 logs reduction of bacteria upon *in vitro* aPDT of VRE treated with a nanomolar concentration of the Au@AgNP@SiO2@Nc-Van and an infection regression and even complete eradication of VRE *in vivo* using infected mice (Zhou et al., 2018).

A selenium nanoplatform (Se@PEP-Ru) was designed with excellent fluorescent properties for imaging bacteria and with high antimicrobial properties (Huang et al., 2017). Zhao and co-workers developed an activated theranostics nanoprobe for near-infrared fluorescence imaging and photothermal therapy of MRSA infections, based on SiO2/PAH-cypate nanosystems modified with PEG and Vancomycin-conjugated poly(acrylic acid) molecules (PAAPEG-Van). This probe is activated by bacteria-responsive polyelectrolyte dissociation from silica NPs. The authors believe that this concept can be used as an approach to design and for production of bacteria responsive multifunctional nanomaterials and constitute their ultimate functions in the treatment of drug-resistant bacterial infections (Zhao et al., 2017). Kuo and collaborators developed a nanotheranostics system using Au nanorods conjugated with a hydrophilic photosensitizer, toluidine blue O, that acted as dual-function agents in photodynamic inactivation and hyperthermia against MRSA (Kuo et al., 2009).

## Clinical translation

At present, there are a few metal NPs-based strategies against bacterial infections undergoing clinical trials. The costs associated to the use of nanotechnology platforms are very high, and therefore conventional treatments are preferred. However, these platforms might be preferable in specific situations, with direct impact on the quality of patients life (Caster et al., 2017).

Bio-kil® [3-(Trimethoxysilyl) propyloctadecyldimethyl ammonium chloride] (Cargico Group, Taiwan) is a patented technology that is based on affixing nano-sized antimicrobials onto a large surface area through covalent chemical bonding to form a durable polymer. Bio-kil® eliminates microorganism through a physical biocide process. This type of nanomaterial consists in inorganic metal components and organic quaternary ammonium components. Recently, Bio-Kil® has been shown to reduce the environmental bacterial burden and MDR organisms (Lee et al., 2017).

AgTive (NCT00337714) is a silver-impregnated central venous catheter and has been marketed with the claim to improved bactericidal activity. AgTive catheters are made of polyurethanes impregnated with silver NPs, and their interaction with body fluids and intravenous solutions results in the release of significantly larger amounts of silver ions from the catheter reducing bloodstream infection (Antonelli et al., 2012).

Acticoat is a nanocrystalline silver dressing that acts as an antimicrobial topical, releasing silver into the wound. This nanoformulation has been shown to inhibit *in vitro* biofilms formation in *P. aeruginosa* and *Acinetobacter baumannii* by more than 90% (Potgieter and Meidany, 2017). Madigan Army Medical Center is studying the efficacy of a silver NPs gel SilvaSorb (NCT00659204) and currently is in phase III of the clinical trials. The aim of this study is to compare the antimicrobial efficacy of a one-time application of SilverSorb (AcryMed, Inc., Portland) against the standard antibacterial hand gel Purell (GoJo Industries, Akron), in reducing transient bacterial counts isolated from the hands of 40 patients seeded with *S. marcescens*.

Nano Silver Fluoride is a new formulation that combines silver NPs, chitosan and fluoride and was developed with antimicrobial properties. This nanoformulation has excellent results as antibacterial agent *against S. mutan*s and *Lactobacilli*. Currently, is used to prevent dental caries in children (Dos Santos et al., 2014).

Despite this review do not concern liposomal formulations since it refers to clinical translation other formulations involving NPs, such as liposomal formulations, have been also identified as antimicrobial agents. Most of these formulations rely on the incorporation of traditional antibiotics into nanoliposomes to improve distribution and circulation times (Caster et al., 2017). Table 2 summarizes antimicrobial liposomes, which are undergoing clinical trials. For example, Amikacin (NCT01315691) is a potent aminoglycoside antibiotic that is useful for the treatment of MDR Gram-negative bacteria. Arikace is an inhaled liposomal formulation that encapsulates amikacin composed of dipalmitoyl-phosphatidylcholine (DPPC) and cholesterol (Meers et al., 2008). These formulation have high drug loading and stability when administrated and in phase II trial, there was no notable difference in toxicity between liposomal drug treatment and placebo (Clancy et al., 2013). Another two-inhaled liposomal formulation are currently in clinical trials. Linhaliq (NCT02104245) is a combination of liposomal and aqueous phase ciprofloxacin, whereas Lipoquin (NCT00889967) is a liposomal ciprofloxacin that allows prolonged drug release. Both of these nanoformulation were developed for the treatment of non-cystic fibrosis bronchiectasis (NCFBE) patients with chronic lung infections with *P. aeruginosa*. Phase II in patients with both CF and non-CF bronchiectasis have been completed. After analysis of clinical data from the two different formulations, Linhaliq showed better performance. The Food and Drug Administration (FDA) has designated Linhaliq as a qualified infection disease product and made it eligible for Fast track designation. In 2016, Pulmanic completed two phases III clinical trials, but has not yet been approved by the FDA. The Hadassah Medical Organization (Jerusalem, Israel) has incorporated quaternary ammonium polyethyleneimine (QA-PEI) based polymers into dental composites. The bacterial membrane may be disturbed by the charged quaternary moiety, it also has potent activity against a series of Gram-positive and -negative pathogens (Ortega et al., 2015). In 2013, these nanoformulation completed phase II trials but no data on outcome have been released to date.

**Table 2:** Antimicrobial liposomal nanoformulation in clinical development.

**Name**	**Antimicrobial**	**Clinical trial phase**	**Target pathogens**	**ClinicalTrials.gov Identifier**
Arikace	Amikacin	III	Gram-negative bacteria	NCT01315691
Lipoquin	Ciprofloxacin	II	Gram-negative bacteria	NCT00889967
Pulmaquin	Ciprofloxacin	III	Gram-negative bacteria	NCT02104245
Silvasorb	Silver	III	Gram-negative bacteria	NCT00659204
MAT2501	Amikacin	–	Gram-negative bacteria	–
QA-PEI	Ammonium Polyehtyleneimine	I-II	Gram-negative and -positive bacteria	NCT01167985

MAT2501 is designed to targeted delivery of the antibiotic amikacin while providing an improved safety and tolerability profile. Currently, Matinas Biopharma has reported positive data from the Phase I study in healthy volunteers for the treatment of MDR Gram-negative bacterial infections and is in preparation for a phase II in patients.

## Other potential applications of NPs

In the case of non-antibiotic therapy, combinations of NPs with essential oils, peptides and other natural compounds have featured as promising antimicrobial strategies. The therapeutic applications of these substances are often limited by their toxicity and volatility (Chen F. et al., 2009; Allahverdiyev et al., 2011). A recent study has shown that chitosan NPs vectors, modified with eugenol and carvacrol essential oils on their surface, were active against *E. coli* and *S. aureus* at concentrations better or equal to unmodified NPs versions (Chen F. et al., 2009). Furthermore, the toxicity of the conjugates toward mouse fibroblasts was significantly less than the pure oils alone. With regards to peptides, the active sequences can be vulnerable to denaturation, aggregation or hydrolysis within end products or in the human body. Colloidal systems containing NPs are at the forefront of peptide research, as they can be designed to encapsulate and protect peptides during biological transit. Water in oil micelles have been successfully used to increase the potency of antimicrobial peptides against *E. coli* (Gontsarik et al., 2016). In another example, liposomes have been used to improve the stability of encapsulated nisin against pH and temperature extremes thereby increasing its potential in food processing (Taylor et al., 2007). Popular NPs vehicle materials for peptides include phytoglycogen NPs (Bi et al., 2011), chitosan (Wu et al., 2017), pectin (Krivorotova et al., 2017), and alginate (Khaksar et al., 2014).

NPs have also been applied with tremendous success in biodetection systems, namely as sensors and diagnostics platforms with increased sensitivity and selectivity. Due to the decrease in size of the transduction mechanisms provided by NPs, most of these platforms have found applications at point-of-need and/or point-of-care (Costa et al., 2014; Veigas et al., 2014; Weng et al., 2015; Kim J. et al., 2017; Wang et al., 2017b; Galvan and Yu, 2018; Yang et al., 2018). In some cases, diagnostics/sensing and therapeutic properties have been combined onto single NPs, providing for innovative tools – Nanotheranostics. Recently, several nanotheranostics strategies against bacteria have been described (Kuo et al., 2009; DeGrasse, 2012; Dai X. et al., 2013; Khlebtsov et al., 2013; Kim et al., 2013; Gamella et al., 2014; Pei et al., 2014; Setyawati et al., 2014; Patel and Janjic, 2015; Thompson et al., 2015; Jagtap et al., 2017; Mocan et al., 2017; Zhao et al., 2017).

## Bottlenecks and future challenges of NPs

Despite the foreseen potential of NPs for medical applications, there are still several bottlenecks related with their acute and long-term exposure in humans. Several routes of exposure must be considered when evaluating NPs exposure, such as oral and gastrointestinal tract, dermal, respiratory system, and endovenous administration directly to the bloodstream (De Matteis, 2017). It is well known also that the physicochemical properties of NPs (e.g., size, shape and surface chemistry) affect their interaction with biological systems, influencing cellular uptake, pharmacokinetics, biodistribution, all of them with direct impact on final biological effects (for recent reviews see Bakand and Hayes, 2016; Xia et al., 2016; De Matteis, 2017; Warheit, 2018). These aspects have been addressed over the past years *via* the evaluation of the *in vitro* and *in vivo* toxicity of metal and metal oxide NPs (Dobrovolskaia et al., 2007; Asharani et al., 2010; Li et al., 2010; Baek and An, 2011; Hackenberg et al., 2011; Conde et al., 2012, 2014; Bondarenko et al., 2013; Ivask et al., 2014; Larsen et al., 2016; Sukwong et al., 2016; Rai et al., 2017), whose conclusions concerning their nanosafety differ depending on the type of assessment. This poses a major concern to effectively draw critical conclusions on NPs safety due to the vast number of different types/shapes/surface modified nanoparticles, the different methods used to evaluate their safety and environmental effects, and also by the fact most of these *in vitro*/*in vivo* studies present acute studies rather than long-term exposure (Bakand and Hayes, 2016; Xia et al., 2016; De Matteis, 2017; Warheit, 2018). Nevertheless, these *in vivo* and *in vitro* studies have been providing clues to the specific mechanisms by which NPs trigger an adverse effect enabling future surface modification of NPs to make them safer and less toxic (De Matteis, 2017). These concerns relating to nanosafety have been addressed and implemented *via* European Commission FP7 and H2020-sponsored programs followed by some relevant conclusions issued by the US National Academy of Science Committee on Research Progress of Environmental Health and Safety Aspects of Engineered Nanomaterials (Warheit, 2018).

Due to the 3Rs (Replacement, Reduction and Refinement) policies of *in vivo* studies, the future challenge of Regulatory Agencies is the standardization of the *in vitro* methodologies to establish the toxicology profile of NPs based on good laboratory practice (GLP) and the construction of flexible and reliable databases in which NPs are classified according to the data derived from these toxicological investigations. Together, these efforts might provide information on the dosage at which a particular NP can be considered safe and thus appropriate for medical use.

## Challenges of current research

As mentioned above, nanomaterials have great potential to prevent and treat bacterial infection, but several challenges remain for the translation to the clinics. Some of these include assessing the interactions of nanoantibiotics with cells, tissues and organs, for dose recalibration and identification of appropriate routes of administration (Sandhiya et al., 2009). The biocompatibility of NPs is generally evaluated through *in vitro* assays, using cell culture. Because NPs, used as antimicrobial agents can enter through skin contact, ingestion, inhalation, oral and intravenous injection, *in vivo* models must also be applied to better understand their effects, including potential toxicity, clearance and metabolism (Beyth et al., 2015). Several studies have shown that intravenously injected NPs accumulate in the colon, lung, bone marrow, liver, spleen and lymphatics (Hagens et al., 2007). Inhalation has also been shown to cause cytotoxicity at the lung, and in the liver, heart and spleen through systemic circulation (Poma and Di Giorgio, 2008; Leucuta, 2013). This is of particular relevance for small NPs because of efficient cellular uptake and transcytosis across epithelial and endothelial cells into the blood and lymphatic circulation. Several NPs systems have demonstrated toxicity in multiple organs, such as free radical-mediated oxidative stress generated by the interaction of antimicrobial NPs with cell components that can result in hepatotoxicity and nephrotoxicity (De Jong and Borm, 2008; Lei et al., 2008).

The effective translation to the clinics will require appropriate guidelines for production and scale-up of manufacturing these nanomaterials, for characterization of the physico-chemical properties and their impact on biocompatibility, for standardization of nanotoxicology assays and protocols to assist easy comparison of data originating from *in vitro* and *in vivo* studies, for the evaluation of their metabolism and mode of action (Duncan and Gaspar, 2011; Bertrand and Leroux, 2012; Beyth et al., 2015; Cordeiro et al., 2016; Rai M. et al., 2016; Zazo et al., 2016). Finally, the community still needs to address the economic impact of translation of these nanomaterials to the clinics.

## Conclusions

Given their vast therapeutic potential, it is becoming increasingly important to understand the mechanisms by which NPs complexes can impact bacterial viability. While one of the beneficial aspects of NPs drug carriers involves “macro-targeting,” *i.e*., specific delivery to the site of infection, understanding the “micro-targeting” of bacterial mechanisms is imperative for the widespread future use of these vectors. Their impact of cell functions such as cell wall permeability, efflux activity, formation of reactive species, and inhibition of essential cellular metabolism and reproduction is of utmost importance.

## Author contributions

PB supervision and correction of Nanoparticles part of the manuscript; MPM supervision and correction of MDR bacteria part of the manuscript; AC draft Nanoparticles part of the manuscript, figure draw, tables design; DF draft MDR bacteria part of the manuscript; NM draft MDR bacteria part of the manuscript; MM coordination of MDR bacteria part of the manuscript and final correction and integration; AF coordination of Nanoparticles part of the manuscript and final correction and integration.

### Conflict of interest statement

NM was employed by the company Nuritas limited. The remaining authors declare that the research was conducted in the absence of any commercial or financial relationships that could be construed as a potential conflict of interest.
